# Fear of Coronavirus, Stress and Fear of Childbirth in Polish Pregnant Women during the COVID-19 Pandemic

**DOI:** 10.3390/ijerph182413111

**Published:** 2021-12-12

**Authors:** Joanna Dymecka, Rafał Gerymski, Adrianna Iszczuk, Mariola Bidzan

**Affiliations:** 1 Department of Health Psychology and Quality of Life, Institute of Psychology, Opole University, 45-040 Opole, Poland; jdymecka@uni.opole.pl; 2 Faculty of Health Sciences, Opole University, 45-040 Opole, Poland; adrianna.iszczuk@uni.opole.pl; 3 Department of Clinical and Health Psychology, Institute of Psychology, University of Gdansk, 80-309 Gdansk, Poland; mariola.bidzan@ug.edu.pl

**Keywords:** COVID-19, fear of coronavirus, fear of childbirth, pregnancy, stress, well-being

## Abstract

The COVID-19 pandemic is the largest pandemic of an aggressive coronavirus in the human population in the 21st century. The pandemic may have a negative emotional impact on pregnant women, causing fear and stress. Negative feelings during pregnancy later affect fear of childbirth. Our study aimed to determine the relationship between fear of COVID-19, stress and fear of childbirth. We assume that fear of COVID-19 will be a mediator of the relationship between perceived stress and fear of childbirth. A total of 262 Polish pregnant women participated in this study. Perceived Stress Scale (PSS-10), Fear of COVID-19 Scale (FOC-6) and Labour Anxiety Questionnaire (KLP II) were used in the study. There was a statistically significant, moderate, and positive relationship between perceived stress, fear of COVID-19, and fear of childbirth. Fear of COVID-19 was a statistically significant mediator in the relationship between perceived stress and fear of childbirth. The COVID-19 epidemic may have a negative emotional impact on pregnant women, causing fear, stress and increased fear of childbirth. Childbirth during the COVID-19 pandemic is perceived by women as a threat to their well-being and health. Therefore, it is especially important to support a woman in the perinatal period and to enable her to give birth to a child.

## 1. Introduction

Pregnancy is a time of many changes in a woman’s life. It may be associated with significant emotional stress, which may affect up to 75% of women [1]. Pregnant women are concerned about their health and their unborn child’s health. Negative life events such as the outbreak of a contagious disease can also be the cause of stress. Pandemic brings uncertainty, numerous restrictions and changes, and a significant number of stressors [2].

The COVID-19 pandemic caused by SARS-CoV-2 is the largest pandemic in the 21st century. The clinical course of the COVID-19 disease varies from asymptomatic and mild to severe disease and death [3]. Pregnant women are at increased risk of severe COVID-19. Most women will experience mild disease, but some may require mechanical ventilation and intensive care therapy [4]. In previous SARS-CoV and MERS-CoV coronavirus outbreaks, significant rates of maternal complications, including intensive care admissions, need for mechanical ventilation and deaths have been observed [5]. This may have exacerbated fear in pregnant women during the current pandemic. 

Although it was initially believed that COVID-19 is not associated with a significant risk of severe disease in pregnant women, the same cannot be said for its psychological effects [6]. Pregnant women feel stress, fear and anxiety during the global pandemic, which has caused the death of more than 3 million people worldwide. As a result of the rapidly increasing number of cases and deaths, both at-risk individuals and society as a whole experience psychological distress and other mental health problems [7,8,9,10]. Increased responses to stress during and immediately after a serious life event are associated with adverse effects on health, which is particularly important for pregnant women.

The presence of contagious diseases contributes to the increased fear in society, as has already been demonstrated in previous epidemics [11,12]. Fear is an adaptive protective mechanism for animals and humans that is fundamental to survival and involves several biological processes of preparation to respond to potentially dangerous events. However, when it is chronic or disproportionate, it can cause psychiatric disorders [11]. During the current pandemic, there is a fear of getting infected, of dying and losing a loved one, and of contact with people who might be infected [13,14]. The COVID-19 pandemic probably increased fear among pregnant women. Women are most concerned about their elderly relatives, the health of their children and the health of their unborn child. More than half of women experience significant health anxiety [15]. Studies show that pregnant women are more likely to develop a severe infection than others. Despite many preventive behaviors, most women are still afraid of getting infected [16]. Pregnant women are afraid of both the continuation of pregnancy and the risk of their own life and the need to terminate it as a result of infection. They are also afraid of the transmission of the infection to the fetus, as well as isolation and quarantine [17].

Pregnancy is also a period of preparation for childbirth, which for many women is a difficult and even traumatic situation. During a pandemic, fear of childbirth may be aggravated by a change in the course of labor. Women are afraid of the course of labor, complications, threats to their own and their child’s health and life and, above all, intense pain sensations. The fear of childbirth is also associated with the threat to the health of the child and mother, the possibility of complications, giving birth to a child with a defect, staying in the hospital, unfriendly behavior of the staff, improper behavior during childbirth and lack of control over one’s own behavior, especially shouting. Studies have shown that women who are younger, have a lower level of education, are in a worse financial situation, have previous negative obstetric experiences or complications, or are in need of a cesarean section are characterized by a stronger fear of childbirth than others. Some personality traits are also predisposed to anxiety by a high level of neuroticism, pre-existing anxiety disorders, symptoms of depression, obsessive compulsive disorders and experiencing sexual violence in childhood. The fear of childbirth is also higher in complicated pregnancies. A stronger fear of childbirth is also characteristic of women who assessed their previous birth as traumatic. There are two types of fear of childbirth: primary—fear of childbirth in a woman who has had no previous experiences in pregnancy—and secondary—fear of childbirth developing after a traumatic obstetric event in a previous pregnancy [18,19,20,21,22,23]. 

We suspect that fear of COVID-19 is one of the predictors of fear of childbirth. However, there are currently no studies investigating the relationship between these variables. However, it has been shown that the COVID-19 pandemic caused an increase in fear among pregnant women [14,15], and negative feelings, thoughts and emotions during pregnancy, including early pregnancy, influence the later fear of childbirth [19]. Research shows that fear of childbirth is associated with the severity of general anxiety [20,21], and that anxiety as a trait is its predictor [22]. Studies have also shown that support from loved ones is a mediator of fear of childbirth [22,23,24], while during a pandemic, family births were banned and women were deprived of this direct support. 

Therefore, the aim of the study was to determine the relationship between fear of COVID-19, stress and fear of childbirth and to check whether the fear of COVID-19 is a mediator between stress and fear of childbirth. We assume that the studied variables will be correlated with each other and that fear of COVID-19 will be an important predictor of fear of childbirth and a mediator of the relationship between perceived stress and fear of childbirth.

## 2. Materials and Methods

### 2.1. Participants and Procedure

We decided to verify the proposed research questions in the cross-sectional correlational study. A total of 262 Polish pregnant women participated in this study. The average age of respondents was 28.40 years. Inclusion criteria were the following: (1) being older than 18 years, (2) being a person whose mother tongue is Polish and (3) ability to give informed consent. Exclusion criteria were the following: (1) having a cognitive impairment or the diagnosis of mental illness, (2) being hospitalized in the last 30 days for any psychological or psychiatric reason and (3) inability to fill out questionnaires for any reason. The questionnaires were delivered to the individuals by a trained study assistant. Their exact characteristics are shown in Table 1. Due to the epidemiological threat, the respondents completed the questionnaires via the Internet. The prepared questionnaire was posted on social networks in groups of pregnant women. The study participants were informed about the anonymity of the study. They could stop filling the survey at any time and without giving any reason. All respondents gave their informed consent to participate in this study, which was carried following the guidelines of Opole University’s Ethics Committee (KEBN 15/2021). The research was conducted during the first wave of the COVID-19 pandemic from 15 March 2020, to 30 May 2020. At that time, the number of cases in Poland was not very large, however, there was a total lockdown. Access to health care was limited, family births and visits to hospitals were forbidden.

### 2.2. Measures

Perceived level of stress was measured with the Perceived Stress Scale (PSS-10) [25]. It consists of 10 questions on a 5-point scale, where: 0—“never”; 4—“very often”. It includes questions such as “Over the past month, how often have you felt nervous and tense?”. The original version of the scale shows good reliability (Cronbach’s α from 0.78 to 0.86).

Fear of the coronavirus was measured with the Polish Fear of COVID-19 Scale (FOC-6) [26]. It is a 6-item questionnaire. Respondents answer the questions using a 5-point scale, where: 1—“I strongly disagree”; 5—“I strongly agree”. It includes questions such as “I am afraid of losing my life due to coronavirus infection”. The scale shows good reliability (Cronbach’s α = 0.83).

Fear of childbirth was measured with the Polish Labour Anxiety Questionnaire (KLP II) [27]. It is a 9-item questionnaire. Respondents answer the questions using a 4-point scale, where: 1—“Definitely not”; 4—“Definitely yes”. It includes questions like: “I am afraid that my labour will be painful”. The scale is characterized by acceptable reliability (Cronbach’s α = 0.69).

### 2.3. Statistical Analysis

Group homogeneity was analyzed with *t*-test and one-way ANOVA comparisons. To verify the hypotheses, it was decided to use the correlation analysis and mediation analysis. The significance of the relationships between variables was tested with Pearson’s r correlation. The mediation analysis was performed using the PROCESS v3.4 macro [28].

## 3. Results

### 3.1. Group Homogeneity Analysis 

Before verifying the mediation hypothesis, it was decided to check whether the tested sample of pregnant women is a homogeneous group. For this purpose, the *t*-test and one-way ANOVA were used. The analyses showed that most of the grouping variables did not significantly differentiate the levels of perceived stress and fear of COVID-19. Statistically significant differences in the level of fear of childbirth were be observed for some grouping variables, however, the effect size measures of these differences ranged from small to moderate. Due to the lack of strong effect sizes and large differences in the numbers in individual categories of grouping variables, it was decided to treat the presented group of study participants as homogeneous. More detailed data are shown in Table 2.

### 3.2. Correlation and Mediation Analysis

The analysis of the Pearson’s r correlation showed that there was a statistically significant, moderate and positive relationship between three tested variables—perceived stress, fear of COVID-19 and fear of childbirth. In the next step, a bootstrapped mediation analysis (5000 samples) [29] using the PROCESS 3.4 MODEL 4 was used [28]. Before performing the analysis, variables were z-scored to obtain *Beta* coefficients. Results show that all tested paths were statistically significant. Perceived stress acted as a predictor of fear of childbirth in the tested sample. Furthermore, fear of COVID-19 was a statistically significant mediator in the relationship between perceived stress and fear of childbirth. It is possible that perceived stress might be related to fear of childbirth via fear of COVID-19. More detailed data are presented in Table 3. 

## 4. Discussion

The aim of the study was to determine the relationship between perceived stress, fear of COVID-19 and fear of childbirth in Polish pregnant women during a global pandemic. Analyses point out that the relationship between all tested variables was significant. Perceived stress, fear of COVID-19 and fear of childbirth were positively correlated with each other. Mediation analysis showed that fear of COVID-19 acted as a mediator between perceived stress and fear of childbirth.

Pregnancy is associated with significant emotional stress, which is caused by numerous physiological, physical and psychological changes [30,31,32,33,34,35]. During this time, women are concerned about medical problems, symptoms, changes in the body, childbirth and the health of their unborn child [36,37]. The present study showed that the level of stress measured by the PSS-10 (20.60) is higher compared to the pre-pandemic result of pregnant women (18) [31]. It is also higher than the Polish population average (16,6) [38]. This is consistent with research that shows that pregnant women experience greater stress compared to people in the general population [31]. Some researchers suggest that stress may occur in up to 75% of pregnant women [1].

In addition to factors specific to each pregnancy, important causes of stress include the difficult events experienced during a pregnancy, for example, the pandemic of a contagious disease. Studies show that pregnant women during pandemic are experiencing moderate to high levels of psychological stress [39,40,41]. This is likely a result of the social, economic and health-related complications that affect pregnant women, as well as uncertainties about the effects of COVID-19 on the fetus [42]. COVID-19 restrictions, isolation and staying-at-home orders can adversely affect the functioning of pregnant women and increase symptoms of stress [39]. Moreover, pregnant women in most countries suffer from significant changes in the management of pregnancy, the course of labor and postpartum care. Many health services have reduced face-to-face visits and limited medical services to using telehealth [6].

Recent research identified two major domains of stress related to the COVID-19 pandemic in pregnant women in the US, Poland, Germany, and Israel: stress related to feeling unprepared for the birth due to the pandemic and stress associated with fear of SARS-CoV-2 infection [2,43]. Both types of stress are related to the severity of the fear of coronavirus. One study found that around one-third of pregnant women during the pandemic experienced stress, both about preparing for childbirth and about the risk of infection [42].

The current study showed that the stress experienced by pregnant women is related to fear of COVID-19. Preis et al. [2] found that pregnant women experienced a high prevalence of mild, moderate and severe fear of COVID-19. Other research also found that the COVID-19 pandemic caused an increased fear among pregnant women. Almost half of the women were very afraid of the vertical transmission of the disease [39]. The research shows that pregnant women are afraid of both the continuation of pregnancy and the risk to their own life and the need to terminate it as a result of infection. They are also afraid of isolation and quarantine [17]. Pregnant women believe that they are more likely to develop a severe course of infection and that they can pass the infection onto their unborn child [16].

Additionally, during the current COVID-19 pandemic, the rules of care for a pregnant woman have changed in many countries. Access to health services has been difficult. Prenatal care services have been postponed except in compulsory cases and in emergencies, while in some countries, pregnant women were only asked to come to give birth. The lack of medical appointments and disturbing information from the media also increased fear and uncertainty in pregnant women” [19,44]. Not surprisingly, more than 80% of women expressed fear of childbirth during the COVID-19 pandemic [6].

In the present study, fear of COVID-19 was found to mediate the relationship between perceived stress and fear of childbirth. Negative feelings, thoughts and emotions in pregnancy affect the later fear of childbirth [20]. Pre-pandemic studies showed that fear of childbirth was associated with the severity of general anxiety [21,22] and that anxiety as a trait was its predictor [23]. It has also been shown that women’s experiences related to the fear of childbirth appear to be related to their quality of life and symptoms of stress [20,45]. It has been shown that fear of COVID-19 was negatively related to the quality of life of pregnant women [46]. Additionally, other studies showed that the risk perception associated with fear of coronavirus can increase the level of anxiety and mediate the relationship between social support and anxiety in pregnant women. These studies showed that the higher the level of risk perception, the greater the level of anxiety [47]. Several studies have also shown that fear of COVID-19 can act as a mediator between intolerance to uncertainty and psychological well-being [48] and between perceived health status and mental health, insomnia and preventive behaviors [49].

Pregnant women during the COVID-19 pandemic experience severe stress because they cannot follow the previously prepared birth plan. Some of them are concerned about whether family members are present during childbirth. They may also be worried about whether a woman or someone in her family will be in quarantine. The course of labor during an epidemic is also a cause for concern [17]. Research suggests that inability to make decisions about childbirth and lack of control may be perceived as traumatic. In many countries, women must come to their prenatal visits without accompanying persons, and in some countries (including Poland), family births have been suspended, although it is known that family support during childbirth is considered essential for women’s well-being [50].

Childbirth during the COVID-19 pandemic is perceived by women as a threat. In addition, there may be fewer healthcare workers on maternity wards than there were before the pandemic due to the relocation of staff to infectious departments and infections or quarantine of the staff. Healthcare workers may also limit contact with women because of fear of infection [19,44,50,51,52]. Therefore, many studies indicate increased fear of childbirth caused by the COVID-19 pandemic. One study showed that 16% of patients underwent cesarean section at the request of the mother [39]. This percentage is much higher than the 5-10% rate reported in the literature [53]. During the pandemic, due to new hospital restrictions, expectant mothers will have to go through higher levels of stress and fear as they will now have to cope alone as no spouse or companion is allowed to be in the delivery room to support them [6]. Studies have shown that support from loved ones is a mediator of fear of childbirth [24], but in the COVID-19 pandemic, family births were suspended in many countries, and women were deprived of this direct support. Fear and lack of support were predictors of fear of childbirth [22,35,54]. It has been shown in the literature that the support of the loved one of the pregnant women in the perinatal period is important in reducing fear and stress [41]. Among the human rights relating to pregnancy and childbirth, the WHO recognizes the importance of companionship in childbirth. Unfortunately, the pandemic has drastically changed care for women and children [6].

The presented study is not free from limitations. To verify tested relationships, a longitudinal study should be carried out. Additionally, international research should be carried out to verify the significance of the proposed model. This would additionally require the use of the cultural equivalence analysis to compare various cultural groups. Since the presented results are based on the Polish population, they cannot be extrapolated to other cultural groups. What is more, our study is based on self-report data, which could have caused an undetected bias. Lastly, we did not measure many important variables, which could have influenced our results. Since relational and personal resources play a significant role in dealing with pandemic fear [55], it can be assumed that providing support and care to pregnant women will be a factor that will significantly reduce the fear of childbirth during the COVID-19 pandemic. This limitation should be corrected in future studies on COVID-19 in pregnant women.

## 5. Conclusions

From current research, it can be concluded that the COVID-19 epidemic may have a negative emotional impact on pregnant women, causing fear and stress. Our results show that fear of COVID-19 was a mediator in the relationship between perceived stress and fear of childbirth. It is also worth paying attention to the fact that strong negative emotions that appear during pregnancy may cause and increase pregnancy symptoms and pregnancy complications, and may affect the mother’s well-being, the course of pregnancy and the child’s condition. Many studies have shown that high levels of perceived stress and fear during pregnancy are associated with several negative health consequences such as pregnancy complications, miscarriages, preterm labor, low birth weight, postnatal depression and negative developmental outcomes in infancy [56,57,58,59]. Therefore, it is especially important to support a woman in the perinatal period and to enable her to give birth to a child with companionship.

## Figures and Tables

**Table 1:** Characteristics of the studied sample (NN = 262).

	*M*	*SD*	*Min*	*Max*
Age	28.40	3.78	18.00	39.00
Week of pregnancy	31.58	7.09	7.00	42.00
	*n*		*%*	
Education				
Basic	1		0.38 %	
Vocational	6		2.29 %	
Secondary	68		25.95 %	
Higher	187		71.37 %	
Place of residence				
Village	87		33.21 %	
Town (<100,000 citizens)	73		27.86 %	
City (>100,000 citizens)	102		38.93 %	
Family Childbirth Planning				
Yes	220		83.97 %	
No	42		16.03 %	
Pregnancy				
First	144		54.96 %	
Subsequent—Second	88		33.59 %	
Subsequent—Third	23		8.78 %	
Subsequent—Fourth	4		1.53 %	
Subsequent—Fifth and more	3		1.15 %	
Childbirth				
First	171		65.27 %	
Subsequent—Second	80		30.53 %	
Subsequent—Third	9		3.44 %	
Subsequent—Fourth	1		0.38 %	
Subsequent—Fifth and more	1		0.38 %	
Chronic Diseases				
No	190		72.52 %	
Diabetes	5		1.91 %	
Hypertension	6		2.29 %	
Cholestasis	2		0.76 %	
Hypothyroidism	48		18.32 %	
Other (unspecified)	23		8.78 %	
Trimester				
First	10		3.82 %	
Second	36		13.74 %	
Third	216		82.44 %	

**Table 2:** Results of the *t*-test and one-way ANOVA group comparisons (N = 262).

*Dependent Variable*	Grouping Variable	Group 1		Group 2		*t*_260_	*p*	*d_Cohen_*
		*M*	*SD*	*M*	*SD*			
Perceived Stress	Family Childbirth Planning (1—Yes; 2—No)	20.95	8.05	18.76	8.99	1.59	0.114	0.26
Fear of Coronavirus		23.15	5.68	22.60	5.67	0.58	0.562	0.10
Fear of Childbirth		15.57	5.37	13.48	5.97	2.28	0.024	0.37
Perceived Stress	Pregnancy (1—First; 2—Subsequent)	20.10	8.45	21.21	8.45	−1.08	0.281	0.13
Fear of Coronavirus		23.66	5.13	22.33	5.13	1.90	0.059	0.26
Fear of Childbirth		16.28	4.98	13.96	4.98	3.47	0.001	0.47
Perceived Stress	Childbirth (1—First; 2—Subsequent)	19.97	8.33	21.78	7.96	−1.71	0.089	0.22
Fear of Coronavirus		23.63	5.22	22.00	6.34	2.23	0.027	0.28
Fear of Childbirth		16.37	5.06	13.11	5.72	4.74	<0.001	0.60
Perceived Stress	Chronic Diseases (1—Yes; 2—No)	22.47	8.49	19.89	8.04	2.28	0.023	0.31
Fear of Coronavirus		23.92	4.87	22.74	5.93	1.51	0.133	0.22
Fear of Childbirth		16.69	5.24	14.68	5.52	2.67	0.008	0.37
*Dependent Variable*	Grouping Variable	*F*	*p*	*η* ^2^ *_p_*	Tukey’s HSD			
Perceived Stress	Education	0.74	0.529	<0.01	-			
Fear of Coronavirus		1.24	0.294	0.01	-			
Fear of Childbirth		0.37	0.769	<0.01	-			
Perceived Stress	Place of Residence	1.09	0.337	<0.01	-			
Fear of Coronavirus		1.13	0.325	<0.01	-			
Fear of Childbirth		6.48	0.002	0.05	Towns > Cities & Towns > Villages			
Perceived Stress	Trimester	0.15	0.858	<0.01	-			
Fear of Coronavirus		2.45	0.088	0.02	-			
Fear of Childbirth		0.76	0.469	<0.01	-			

Note: Education—*df*1 = 3; *df*2 = 258; Place of Residence—*df*1 = 2; *df*2 = 259; Trimester—*df*1 = 2; *df*2 = 259.

**Table 3:** Results of the Pearson’s r correlation and PROCESS model 4 mediation analysis (N = 262).

**Correlation**			***M***	***SD***	***X***	***M***	***Y***
*X*—Dependent Variable—Perceived Stress			20.60	8.23	-		
*M*—Mediator—Fear of Coronavirus			23.06	5.67	0.26 ***	-	
*Y*—Independent Variable—Fear of Childbirth			15.24	5.51	0.47 ***	0.43 ***	-
**Mediation**			***Beta***	***SE***	***p***	***LLCI***	***ULCI***
*X*	->	*M*	0.18	0.05	<0.001	0.077	0.274
*M*	->	*Y*	0.32	0.05	<0.001	0.210	0.419
*X(M)*	->	*Y*	0.25	0.04	<0.001	0.178	0.326
Indirect Effect			0.06	0.04	0.002	0.239	0.388

Note: *** *p* < 0.001; skewness and kurtosis of all tested variables did not exceed the range of <−2; 2>.

## Data Availability

The data can be made available from the corresponding author upon reasonable request.
